# Thymoquinone induces apoptosis in temozolomide‐resistant glioblastoma cells via the p38 mitogen‐activated protein kinase signaling pathway

**DOI:** 10.1002/tox.23664

**Published:** 2022-09-29

**Authors:** Ai Mai, Shu‐Wen Ye, Jia‐Yu Tu, Jun Gao, Zhan‐Fang Kang, Qian‐Ming Yao, Wei‐Jen Ting

**Affiliations:** ^1^ Biomedicine Research Centre The Third Affiliated Hospital of Guangzhou Medical University, Guangzhou Medical University Guangzhou China; ^2^ Department of Neurosurgery The Sixth Affiliated Hospital of Guangzhou Medical University, Guangzhou Medical University Guangzhou China; ^3^ Department of Neurosurgery Affiliated Cancer Hospital & Institute of Guangzhou Medical University, Guangzhou Medical University Guangzhou China

**Keywords:** blood‐brain barrier, glioblastoma, resistance, temozolomide, thymoquinone

## Abstract

Temozolomide (TMZ) can cross the blood‐brain barrier (BBB) and deliver methyl groups to the purine (guanine) bases of DNA, leading to mispairing during DNA replication and subsequent cell death. However, increased expression of the repair enzyme methyl guanine methyltransferase (MGMT), which removes methyl groups from purine bases, counteracts methylation by TMZ. We evaluated the anticancer potential of thymoquinone (TQ), a hydrophobic flavonoid that inhibits resistance and induces apoptosis in various cancer cells, both in vitro and in vivo. In vitro experiments showed that compared with the Hs683 and M059J cell lines, U251 cells were more sensitive to TMZ. Compared to U251 cells, U251R cells, a TMZ drug‐resistant strain established in this study, are characterized by increased expression of phosphorylated extracellular signal‐regulated kinase (p‐ERK) and MGMT. TQ treatments induced apoptosis in all cell lines. The p38 mitogen‐activated protein kinase signal pathway was mainly activated in U251 and U251R cells; however, p‐ERK and MGMT upregulation could not suppress TQ effects. Furthermore, si‐p38 pretreatment of U251R cells in TQ treatments inhibited cell apoptosis. We speculate that TQ contributed to the phosphorylation and activation of p38, but not of ERK‐induced apoptosis (irrespective of TMZ resistance). In vivo, U251R‐derived tumors subcutaneously inoculated in nude mice exhibited significant tumor volume reduction after TQ or TQ + TMZ cotreatments. High‐performance liquid chromatography assay confirmed the presence of TQ in murine brain tissues. Our findings demonstrate that TQ can effectively cross the BBB and function alone or in combination with TMZ to treat glioblastoma.

## 1 BACKGROUND

Glioblastoma (GBM) is a primary malignant brain tumor that has a poor prognosis and an unfavorable overall survival (5‐year survival rate of <7%).^1^, ^2^ Owing to its high‐degree of malignancy, the overall survival rate of GBM patients is not related to tumor location; the prognosis of patients undergoing total resection is better than that of patients with near‐total resection. The current clinical treatment standards for GBM recommend active treatments, such as partial or total resection, as much as possible. However, the patient's quality of life postsurgery may be affected by the location of the total tumor resection.^3^, ^4^ In addition, chemoradiation therapy is recommended, wherein a daily dose of two grays of radiotherapy, combined with temozolomide (TMZ), is administered for 30 days.^5^ Although conventional treatments, including maximum surgical resection, chemotherapy, and radiation therapy with TMZ, are currently available, the survival rate for GBM patients remains very low (approximately 15 months).^6^, ^7^

Temozolomide is one of the few drugs that can be used to treat GBM, mainly because it can pass through the blood‐brain barrier (BBB) and inhibit cell alkylation, thereby inhibiting GBM development. However, many GBM cells with high MGMT expression are not sensitive to TMZ treatment; MGMT expression can be increased through the rat sarcoma virus (RAS)/rapidly accelerated fibrosarcoma (RAF)/extracellular signal‐regulated kinase (ERK) signaling pathway, leading to TMZ resistance.^8^ Mitogen‐activated extracellular signal‐regulated kinase (MEK1) feedback has a variety of compensatory effects, leading to the incomplete inhibition of the RAS/RAF/ERK pathway. A combination of trametinib (MEK1/2 inhibitor) and GDC‐0623 (MEK1 inhibitor) is required for the clinical inhibition of RAS/RAF/ERK.^9^ Recently, clinical studies have investigated the use of TMZ in combination with other drugs, such as bevacizumab (an antivascular endothelial growth factor) and everolimus (a mammalian target of rapamycin [mTOR] inhibitor); however, no changes in survival rates were reported.^10^, ^11^ Therefore, the development of antitumor drugs that can pass through the BBB remains a key target for GBM treatment.

Thymoquinone (TQ) is a hydrophobic flavonoid compound that inhibits resistance and induces apoptosis in various cancer cells.^12^ Our previous studies have shown that TQ presents excellent anticancer effects against oral squamous carcinoma via p38β inhibition; it also exhibits strong tumor suppression ability for other tumors, such as colorectal cancer and hepatocellular carcinoma, through activated p38 signaling.^13^, ^14^ A recent study revealed the expression of a small GTPase, RND2, which functioned as an endogenous repressor of the p38 mitogen‐activated protein kinase (MAPK) and a cause of GBM proliferation.^15^ Moreover, p38 MAPK activation enhanced epidermal growth factor receptor (EGFR) degradation and reduced tumor development.^16^ Based on these considerations, in this study, we hypothesized that TQ might treat GBM through p38 MAPK activation, although it is not currently included in a clinical trial for the treatment of tumors.

In vitro experiments on GBM cell lines (M059J, Hs683, U251, and U251R [a U251‐TMZ resistant cell line]) were used to test the anti‐GBM effects of TQ. We assessed cell viability (MTT assay), protein expression (western blotting assay), and cell apoptosis (DAPI/TUNEL staining assay). U251R cells were also inoculated into nude mice for in vivo experiments to examine whether TQ treatment could induce tumor apoptosis. Furthermore, brain tissue samples of nude mice in TQ and TQ‐TMZ cotreatment groups were analyzed using high‐performance liquid chromatography (HPLC) to investigate whether TQ could enter the brain by passing through the BBB.

## 2 METHODS

### 2.1 Reagents and cell lines

Thymoquinone (274666, Sigma Aldrich, St Louis, Missouri) was dissolved in dimethyl sulfoxide (DMSO) and diluted to 100 mM as a stock solution. TMZ (10 mM in DMSO) and SB203580 (10 mM in DMSO) were purchased from Med Chem Express LLC (Shanghai, China). The p38 (sc‐29433) and ERK (sc‐29307) small interfering ribonucleic acids (siRNAs) were purchased from Santa Cruz Biotechnology Co., Ltd. (Shanghai, China). All chemical reagents were freshly prepared and diluted from stock solutions to the indicated concentrations as working solutions. The U251, M059J, and Hs683 cell lines were obtained from the National Collection of Authenticated Cell Cultures (Shanghai, China). The U251R cells were established from U251 cells using the following procedure. First, 1 × 10^6^ U251 cells in a 10 cm dish were cultured with the DMEM medium containing TMZ (100 μM) for 24 h. Then, the cells were removed from the medium and washed with phosphate buffered saline (PBS); subsequently, the cells were placed again in the normal DMEM culture medium for 48 h or until the cell number recovered to 1 × 10^6^. The above steps were repeated for 10 times, until the cells, for the most part, did not decrease after 24 h of TMZ (100 μM) treatment. Then, portions of these cells were used to detect the half‐maximal inhibitory concentration (IC_50_) of TMZ treatment. According to a report, when the cells undergo TMZ treatment for 24 h and exhibit an IC_50_ > 200 μM, U251 cells are considered resistant to TMZ.^17^ Next, these TMZ‐resistant U251 cells were seeded in a 96‐well plate via serial dilution, and a single TMZ‐resistant cell (U251R) was selected and cultured in DMEM medium containing TMZ (100 μM) until the cell number reached 1 × 10^6^ in a 10 cm culture dish. These U251R cells were used in this study. Moreover, doses of the reagents used for TMZ, TQ, SB203580, U0126, si‐p38, and si‐Erk varied according to our previous research reports and literature review.^13^, ^14^, ^17^

### 2.2 Cell culture

The GBM cell lines, including M059J (which is a radiosensitive line caused by a defect in DNA‐dependent protein kinase and a truncating ataxia telangiectasia mutation),^18^ Hs683 (which is reported with mitogen‐activated protein kinase‐interacting kinase 1 overexpression),^19^ and U251(which has reported MSH6 mutations that may be associated with TMZ resistance during the TMZ treatments),^20^ were cultured in Dulbecco's Modified Eagle's Medium (DMEM, D7777, Sigma Aldrich, St. Louis, Missouri) with 10% fetal bovine serum (FBS). U251R cells were cultured in a normal culture medium with 10% FBS and 50 μM TMZ. All cells were maintained at 1 × 10^5^ cells cm^−2^ in culture dishes with a diameter of 100 mm; the cultured medium was changed every 48 h until the necessary subculture was processed.

### 2.3 Animals

A total of 60 BALB/c‐nu male mice (30.0 ± 0.5 g) were purchased from the Southern Medical University Experimental Animal Center (Guangdong, China); the experimental designs and protocols used in this study were reviewed and approved by the Institutional Animal Care and Use Committee (IUCAC) of Guangzhou Medical University (2019‐740, Guangdong, China). All BALB/c‐nu mice were kept at standard conditions, with a 12 h light/dark cycle, temperatures of 24–26°C, and 60%–70% humidity; normal diets and water were administered ad libitum. In the first step of tumor treatment experiments, U251R cells were prepared in a matrix mixture (50% wt/wt Matrigel in DMEM) and xenografted to each mouse (1 × 10^6^ cells in 0.5 ml) under the back skin. On the 30th day, tumors with diameters >6 mm were considered for the next step (48 mice passed this selection; 12 were excluded). The 48 selected BALB/c‐nu mice were randomly divided into four groups (*n* = 12 each). The control group mice were treated with PBS through intraperitoneal (IP) injections (1 ml kg^−1^ d^−1^). Mice in the TQ treatment group were treated with TQ through an IP injection (50 mg kg^−1^ d^−1^ in PBS), whereas those in the TMZ treatment group were treated with TMZ through an IP injection (50 mg kg^−1^ d^−1^ in PBS); those in the TQ‐TMZ cotreatment group were treated with a TQ and TMZ mixture through an IP injection (50 mg TQ and 50 mg TMZ kg^−1^ d^−1^ in PBS). After treatment for 30 days, all mice were sacrificed using CO_2_, and tumor tissues were immediately collected for further analysis.

### 2.4 Cell viability

In this study, M059J, Hs683, U251, and U251R cells were seeded into 24‐well plates for 4 h and were then placed in a serum‐free medium for another 12 h. Next, all solutions in the culture plates were changed to normal media containing 0, 6.25, 12.5, 25, 50, and 100 μM of TMZ or TQ for 24 h. Then, the cell viability was evaluated using a 3‐(4, 5‐dimethylthiazolyl‐2)‐2, 5‐diphenyltetrazolium bromide (MTT) assay.^13^, ^14^ In brief, cells were incubated in 5 mg ml^−1^ MTT (Sigma Aldrich, St Louis, Missouri) in PBS solution for 4 h after the indicated treatments; cells were then washed in PBS. At the final step, the cellular MTT formazan products in each well were dissolved in DMSO, and the absorbance at 490 nm was measured (SPEXTROstar Nano, BMG LABTECH GmbH, Ortengberg, Germany). Results were used to calculate IC_50_ values for TQ or TMZ after 24 h for every cell line. The 24‐h IC_50_ of TQ for each cell line was used to evaluate the 72‐h cell viability for each cell line (M059J, Hs683, U251, and U251R). Initially, the IC_50_ (estimated value) was calculated, according to the MTT assay to detect the cell viability after 24 h at different doses (0–200 μM) of TMZ. Subsequently, a graph of cell viability against drug concentration was plotted based on the MTT results. Then, a linear calibration curve was generated based on this graph. This calibration curve was used to calculate the approximate concentration at which the cell viability was 50%. Due to the possible influence of factors such as different drugs and cell types, the calibration curve was not linear. Therefore, it became necessary to increase or decrease some drug concentrations based on the IC_50_ value estimated in the second step; following this, the MTT assay (the initial step) was repeated to determine the precise IC_50_ value.

### 2.5 Protein expression and inhibition analysis

U215 and U251R cells were treated with the indicated reagents only, or with the following different combinations: TQ (20 μM), TMZ (100 μM), U0126 (10 μM), or SB203580 (10 μM), for 24 h. The siRNA, including si‐ERK and si‐p38, were dissolved in ribonuclease (RNAase)‐free water and then added to the transfection reagent (PureFection™, System Biosciences, Palo Alto, CA) and serum‐free medium. They were then incubated for 15 min at 25°C before being added to U251R cells. After 48 h of incubation with si‐ERK (10 μM) and si‐p38 (10 μM), the U251R cells were treated with or without TQ (20 μM) and TMZ (100 μM) for 24 h. The proteins expressed from the cells were collected using a protein extraction solution (PRO‐PREP™, iNtRON BIOTECHNOLOGY, Korea) at 4°C. Then, proteins were further analyzed using western blotting assays.^13^, ^14^ In brief, protein samples were subjected to sodium dodecyl (lauryl) sulfate‐polyacrylamide gel electrophoresis (SDS‐PAGE) at 75 V for 2.5 h. The separated proteins were then transferred onto a polyvinylidene fluoride membrane and blocked using 5% bovine serum albumin in Tris Buffered Saline with Tween (20 mM Tris–HCl, 150 mM NaCl, and 0.1% Tween‐20) solution. The expression of target proteins was assessed using the following primary antibodies: ERK (#4695), p‐ERK (#4370), p38 (#8690), p‐p38 (#4511), MGMT (#86039), cleaved caspase‐3 (# 9664), and glyceraldehyde 3‐phosphate dehydrogenase (GAPDH; #5174). All antibodies were purchased from Cell Signaling Technology, Inc. (Danvers, Massachusetts). Finally, images were obtained using a ChemiDoc Imaging System (BioRad, Hercules, California) after 30 min of incubation with an anti‐rabbit immunoglobulin horseradish peroxidase‐linked antibody (#7074, Cell Signaling Technology, Inc., Danvers, Massachusetts). Samples were visualized via chemiluminescence enhanced by the Immobilon Western Chemiluminescent HRP Substrate (p90719, Millipore Corporation, Billerica, Massachusetts).

### 2.6 Apoptosis detection in slices

Tumor tissue samples were freshly collected at the last step of the animal experiment. All tissues were embedded and sliced with the optimum cutting temperature (OCT) compound at −22°C. Slices were then washed twice with PBS to remove the OCT, incubated with 0.1% Triton X‐100 (in PBS) for 10 min, re‐washed twice, and incubated for 60 min in terminal deoxynucleotidyl transferase dUTP‐mediated nick‐end labeling (TUNEL; In Situ Cell Death Detection Kit, POD, Roche Diagnostics GmbH, Mannheim, Germany) at 37°C. Then, 4,6‐diamidino‐2‐phenylindole (DAPI, 0.1 μg ml^−1^) was applied onto tissue slices for 15 min and then washed twice with PBS. The nuclei in the tissue fluoresced at 454 nm, whereas TUNEL‐positive nuclei fluoresced at 460 nm. All tissue slices were imaged using a Zeiss Axiophot microscope (Jena, Germany) for further analysis.

### 2.7 TQ analysis in mouse brain tissue samples

Thymoquinone was analyzed in BALB/c‐nu mice brain tissue samples using HPLC assays.^21^ To this end, 100 mg of the brain tissue from each BALB/c‐nu mouse was freshly collected (within 1 h after the final indicated IP‐injection treatment). These brain tissue samples were then homogenized in 1 ml of hexane and centrifuged at 10 000× g for 10 min at 4°C. The supernatants were further dried in pure dehydrated nitrogen air at 4°C, following which they were re‐dissolved in 1 ml of methanol, filtered with a 0.22‐μm filter, and then immediately analyzed using HPLC. The following HPLC (CBM‐20A, Shimazu, Japan) analysis conditions were used: C‐18 column (150 × 4.6 mm^2^, Kanto Mightysil RP‐18 GP, Japan), mobile phase comprising 70 vol% 60 mM phosphoric acid aqueous solution and 30 vol% methanol (pH 3.5), and flow rate of 1.0 ml min^−1^. Absorbance was detected at 294 nm using an ultraviolet‐detector (SPD‐20A, Shimazu, Japan). The calibration curve was constructed using the HPLC analysis results associated with the brain tissue extracted from control group mice (with 0, 5, 10, and 20 μM of TQ).

### 2.8 Statistical analysis

The results are presented as mean ± *SD*, with at least three independent repeats per sample. Statistical analysis was performed by one‐way analysis of variance (ANOVA) and the Tukey's test. Differences were considered significant at *p* < .05, *p* < .01, and *p* < .001.

## 3 RESULTS

### 3.1 Anti‐cancer effects of TMZ and TQ

**FIGURE 1:**
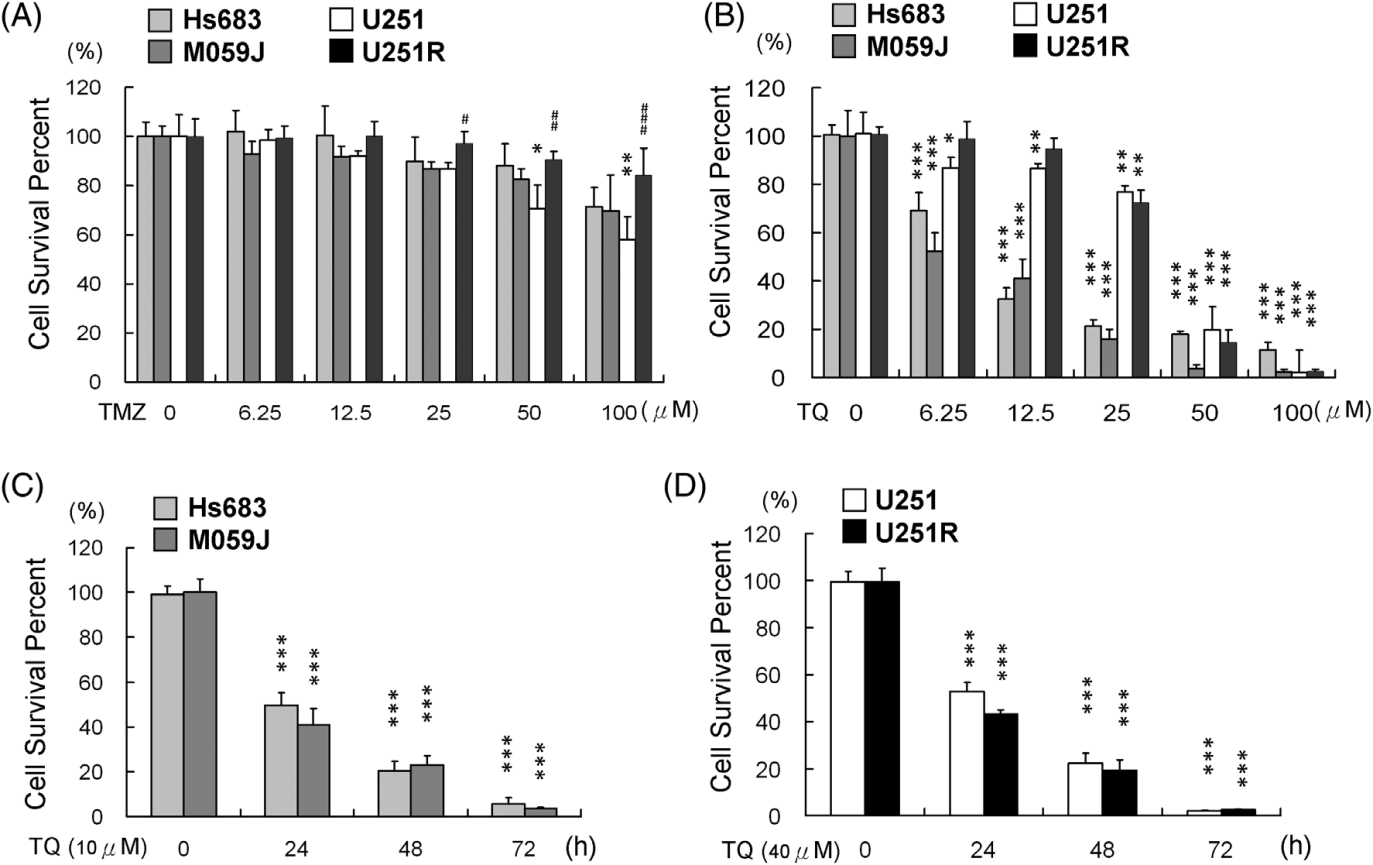
Effect of temozolomide (TMZ) and thymoquinone (TQ) treatment on human glioblastoma cell lines. The viabilities of Hs683, M059J, U251, and U251R 24 h after (A) TMZ treatment. Results revealed that the U251 cell line is very sensitive to TMZ. The viabilities of Hs683, M059J, U251, and U251R 24 h after (B) TQ treatment. All cell lines were more sensitive to TQ than to TMZ. (C) The viabilities of Hs683 and M059J cells 72 h after TQ treatment (10 μM). (D) The viabilities of U251 and U251R cells 72 h after TQ treatment (40 μM). (*p < .05, **p < .01, ***p < .001, compared with U251 control cells; #*p* < .05, ##*p* < .01, ###*p* < .001, compared with U251R control cells)

The anticancer effects of TMZ for each GBM cell line are demonstrated by the cell viability results from the MTT assays, shown in Figure 1A. The IC_50_ values for each cell line were >100 μM. In U251 cells, the 50 and 100 μM TMZ treatments reduced viability by almost 20% and 40%, respectively. When TMZ concentration increased to 100 μM, this caused apoptosis of 20%–30% of Hs638, M0591, and U251R cells. TQ treatments significantly reduced the viability of Hs683, M059J, U251, and U251R cells within 24 h. The 24 h TQ treatment IC_50_ values were 9.4 μM in Hs683 cells, 11.3 μM in M059J cells, 37.5 μM in U251 cells, and 35 μM in U215R cells (Figure 1B). The Hs683 and M059J cells neared depletion after treatment with 10 μM TQ for 72 h (Figure 1C) and the U251 and U251R cells, after treatment with 40 μM TQ for 72 h (Figure 1D).

### 3.2 ERK and MGMT are involved in TMZ resistance

**FIGURE 2:**
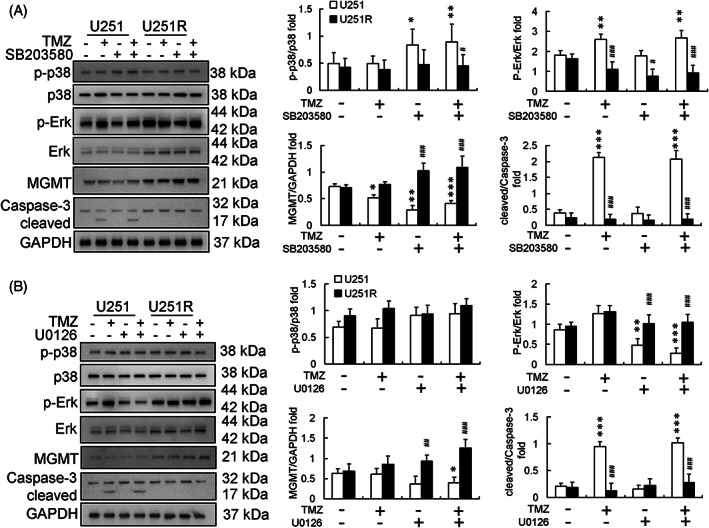
Effect of temozolomide (TMZ) treatment in U251 and U251R cell lines. Protein expression and calibration after pretreatment with (A) SB203580 (10 μM) and (B) U0126 (10 μM) followed by TMZ (100 μM) treatment for 24 h in U251 and U251R cells; both SB203580 and U0126 did not enhance U251R cell apoptosis after TMZ treatment. (*p < .05, **p < .01, ***p < .001, compared with U251 control cells; #*p* < .05, ##*p* < .01, ###*p* < .001, compared with U251R control cells). GAPDH, glyceraldehyde 3‐phosphate dehydrogenase; MGMT, methyl guanine methyltransferase

Treatment with TMZ (100 μM) increased p‐ERK levels rather than p‐p38 levels in both U251 and U251R cells (Figure 2A). TMZ treatment induced apoptosis of U251 cells, irrespective of pretreatment (U0126 [ERK inhibitor], SB203580 [p38 inhibitor], or none). Moreover, TMZ could not induce U251R cell apoptosis with or without U0126 or SB203580 pretreatments (Figure 2B). Pretreatment with U0126 (ERK inhibitor) rather than SB203580 (p38 inhibitor) inhibited TMZ treatment‐induced ERK phosphorylation and downstream MGMT expression in U251 cells (Figure 2B). However, this effect was not observed in U251R cells (Figure 2B).

### 3.3 TQ induces U251 and U251R cell apoptosis through p38 activation rather than through ERK activation

**FIGURE 3:**
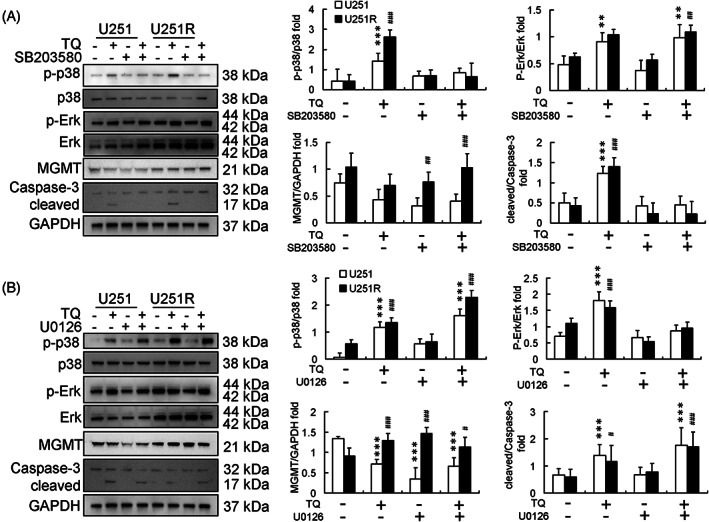
Effect of thymoquinone (TQ) treatment in U251 and U251R cell lines. Protein expression and calibration after pretreatment with (A) SB203580 (10 μM) and (B) U0126 (10 μM) followed by TQ (50 μM) treatment for 24 h in U251 and U251R cells. SB203580 inhibited U251R cell apoptosis and U0126 enhanced U251R cell apoptosis after TQ treatment. (*p < .05, **p < .01, ***p < .001, compared with control U251 cells; #*p* < .05, ##*p* < .01, ###*p* < .001 compared with U251R control cells). GAPDH, glyceraldehyde 3‐phosphate dehydrogenase; MGMT, methyl guanine methyltransferase

Thymoquinone treatments increased p‐ERK and p‐p38 levels in both U251 and U251R cells (Figure 3). SB203580 pretreatment inhibited p‐p38 expression in both U251 and U251R cells in TQ treatments (Figure 3A). Moreover, SB203580 pretreatment blocked TQ‐induced increase in p‐p38 expression in both U251 and U251R cells (Figure 3A). However, U0126 pretreatment induced p‐Erk levels in U251 and U251R cells and enhanced TQ‐induced apoptosis of both U251 and U251R cells (Figure 3B).

### 3.4 TQ‐induced U251R cell apoptosis depends on p38 activation

**FIGURE 4:**
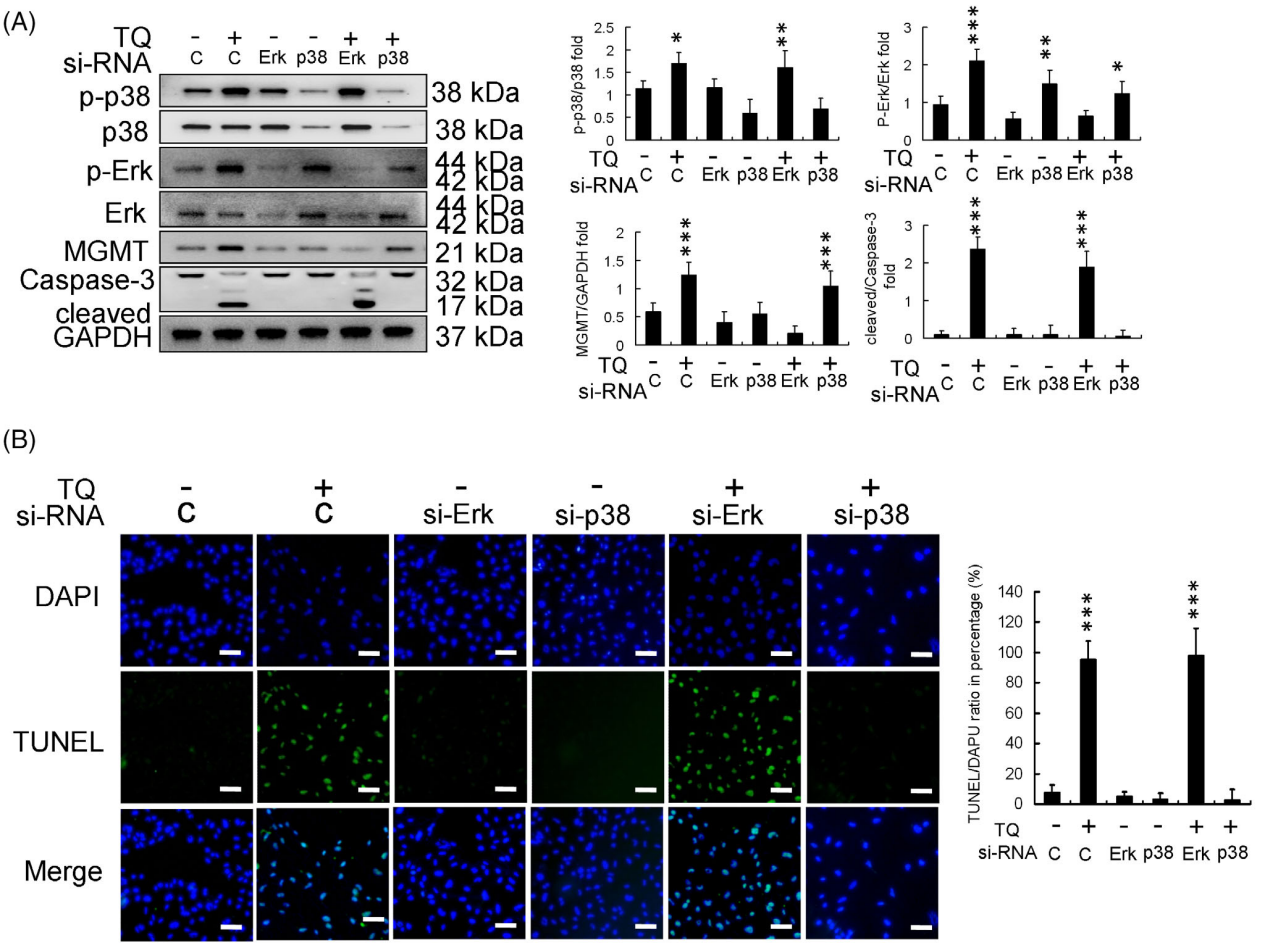
Effect of phosphorylated‐extracellular signal‐regulated kinase (p‐ERK) and p38 protein silencing in thymoquinone (TQ)‐treated U251R cells. (A) Protein expression and calibration. (B) DAPI‐TUNEL staining assay (scale bar in each image: 100 μm) showed that si‐p38 inhibited U251R cell apoptosis and si‐ERK enhanced U251R cell apoptosis in the background of 24 h TQ (50 μM) treatment in U251R cells. (*p < .05, **p < .01, ***p < .001, compared with control). DAPI, 4,6‐diamidino‐2‐phenylindole; ERK, extracellular signal‐regulated kinase; GAPDH, glyceraldehyde 3‐phosphate dehydrogenase; MGMT, methyl guanine methyltransferase; siRNA, small interfering ribonucleic acid; TUNEL, terminal deoxynucleotidyl transferase dUTP‐mediated nick‐end labeling

Methyl guanine methyltransferase expression in the TQ and TQ‐si‐ERK treatment groups was reduced significantly compared with that in the control group. Similarly, the cleaved caspase‐3 expression was higher in the TQ and TQ‐si‐ERK treatment groups. However, in the TQ‐si‐p38 treatment group, the expression of cleaved caspase‐3 was lost (Figure 4A). TUNEL and DAPI staining assays also reveal a higher apoptosis percentage in the responses of the TQ treatment and TQ‐si‐ERK treatment groups; however, apoptotic effects disappeared in the TQ‐si‐p38 treatment group (Figure 4B).

### 3.5 In vivo evaluation of the anticancer effect of TQ

**FIGURE 5:**
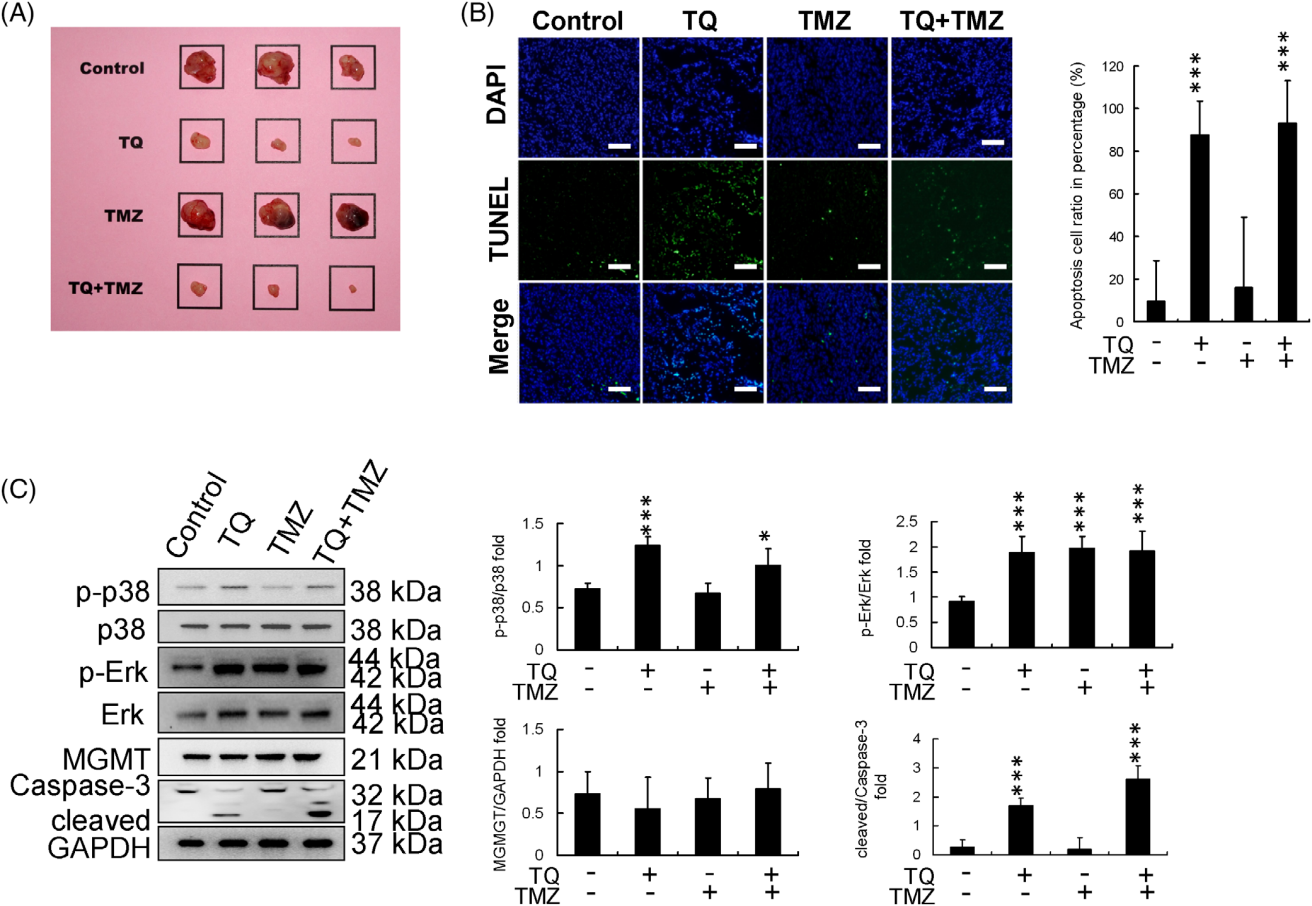
Evaluation of the anticancer effect of thymoquinone (TQ) in vivo. (A) Tumors arising from U251R cells in a mouse xenograft model (for each treatment group); square size is 20 mm × 20 mm. Tumor volumes are reduced in the TQ and TQ + TMZ treatment groups. (B) DAPI‐TUNEL staining assay (scale bar in each image: 100 μm). Apoptotic cell percentage was increased in the TQ and TQ + TMZ treatment groups. (C) Protein expression and calibration in tissue samples. TQ enhanced the levels of p‐p38 and regulated downstream caspase‐3 activation. (*p < .05, **p < .01, ***p < .001, compared with control). DAPI, 4,6‐diamidino‐2‐phenylindole; ERK, extracellular signal‐regulated kinase; GAPDH, glyceraldehyde 3‐phosphate dehydrogenase; MGMT, methyl guanine methyltransferase; p‐ERK, phosphorylated extracellular signal‐regulated kinase; TMZ, temozolomide; TUNEL, terminal deoxynucleotidyl transferase dUTP‐mediated nick‐end labeling

A mouse model featuring U251R cells xenografted to BALB/c‐nu mice was established to evaluate the antitumor effects of the indicated drug treatments in vivo, including TQ, TMZ, and cotreatment with TQ and TMZ. After 30 days of treatment, tumor samples were freshly collected and measured (Figure 5A). The tumor volumes in the TMZ treatment group were similar to those in the control group. In contrast, tumor volumes in the TQ treatment and TQ + TMZ cotreatment groups were significantly reduced (Figure 5A). The tumor tissues were also sectioned at −20°C after OCT compound embedding, and further analysis of apoptosis was conducted using TUNEL and DAPI staining assays. The percentage apoptosis in the tumor samples from each treatment group was also determined (Figure 5B). The apoptotic effects were mild in the TMZ treatment group but substantial in both TQ and TQ + TMZ treatment groups (Figure 5B). Protein levels were also analyzed through western blotting, and the expression ratios were calibrated (Figure 5C). The protein expression results demonstrated that the p‐ERK levels were increased in all the treatment groups compared to those in the control group; however, p‐p38 levels were only increased in the TQ and TQ + TMZ treatment groups, while cleaved caspase‐3 expression in each group paralleled the p‐p38 expression (Figure 5C).

### 3.6 TQ concentrations in mouse brain tissue

**FIGURE 6:**
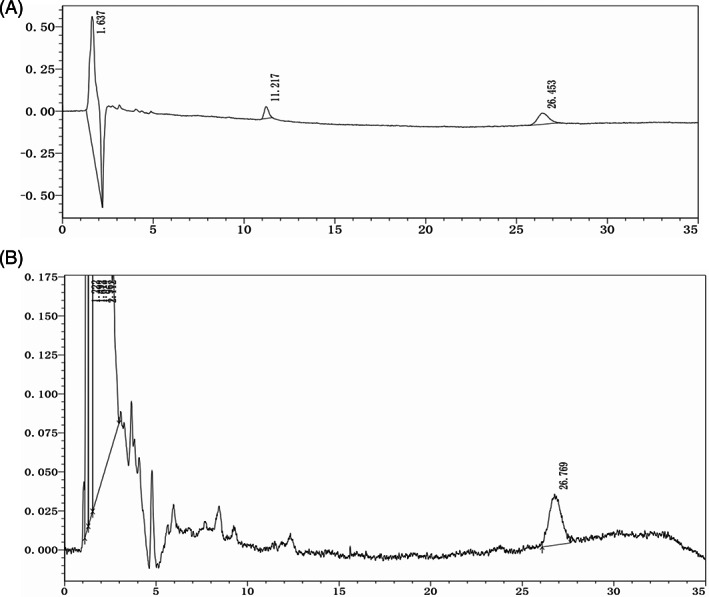
High‐performance liquid chromatography (HPLC). (A) HPLC analysis of 50 μM thymoquinone (TQ) standard solution; TQ retention time: 26.345 min. (B) HPLC analysis of brain extract samples of mice with 1 h TQ intraperitoneal (IP)‐injection; TQ retention time: 26.769 min. The results suggest that TQ exists in mice brain tissue.

High‐performance liquid chromatography of TQ standards (50 μM) revealed that the retention time of TQ was 26.345 min (Figure 6A). However, for mouse brain extract samples, the retention time for TQ was 26.769 min (Figure 6B). On comparing the peak area quantification method to the TQ standard, HPLC revealed that the TQ concentration in the mouse brain tissue was 25 ± 4 μM 1 h after TQ IP‐injection.

## 4 DISCUSSION

In this study, the IC_50_ values for TMZ and TQ treatments of GBM cells—including M059J, Hs683, U251, and TMZ‐resistant U251R cells—were evaluated. Only U251 cells were found to be sensitive to TMZ (Figure 1A). In TQ treatments, M059J and Hs683 cells were more sensitive than U251 and U251R cells (Figure 1B). Furthermore, all cell lines exhibited very low viabilities within 72 h subsequent to TQ treatment at the IC_50_ concentration (Figure 1C,D).

U251R is a single clonal cell line developed from TMZ‐resistant U251 cells (Figure 1A), which exhibited higher MGMT and p‐ERK levels than U251 cells (Figure 2). ERK is the upstream activator protein for MGMT production during the development of TMZ resistance.^22^ In addition to TMZ treatment for GBM, high‐level activation of the RAS/RAF/ERK signaling pathway has been reported as a common drug resistance mechanism in several other cancers, including hepatoma.^23^ Here, SB203850 (p38 inhibitor) and U0126 (ERK inhibitor) were used to evaluate the effects of TMZ treatment in U251 cells. Pretreatment with SB203580 and U0126 did not affect U251 or U251R cells being treated with TMZ (Figure 2). Moreover, the high expression of p‐ERK in U251R cells proved difficult to reduce by U0126 treatment alone (Figure 2B). It has been suggested that, alone or in combination, more potent RAS‐MEK1/2‐ERK blockers are necessary for clinical applications because RAS‐ERK and PI3K‐mTOR crosstalk or other unknown compensation paths for ERK activation might exist.^24^, ^25^, ^26^ Unfortunately, TMZ‐induced apoptosis was not observed in U251R cells, irrespective of U0126 pretreatment (Figure 2B). This may be due to the short‐term treatment of cells with TMZ (24 h) or other effects of TMZ resistance in U251R cells that are still unclear.

TQ treatment induced apoptosis of U251 and U251R cells, regardless of the p‐ERK levels (Figure 3). Pretreatment with SB203580 followed by TQ treatment reduced the apoptotic effects in both U251 and U251R cells (Figure 3A), suggesting that TQ‐induced apoptosis might be regulated by the p38 pathway. Several recent studies have also indicated that p38 MAPK activation is an important phenomenon for inducing human GBM cell apoptosis.^27^, ^28^ Furthermore, p38 MAPK activation was reported to promote internalization and ubiquitin ligase‐mediated degradation of EGFR at the cell surface, thus suppressing proliferation and impairing self‐renewal of GBM stem cells.^16^ Interestingly, unlike TMZ treatment, U0126 pretreatment promoted the apoptosis of U251 and U251R cells after 24 h TQ treatment (Figure 3B).

Here, si‐p38 and si‐ERK treatments successfully reduced p38 and ERK expression in U251R cells (both phosphorylated and non‐phosphorylated forms) in the background of TQ treatment (Figure 4A). In the si‐ERK‐TQ cotreatment group, percentage apoptosis of U251R cells was higher compared to that observed in TQ‐treated cells (Figure 4B). The si‐p38‐TQ cotreatment group showed no apoptosis of U251R cells, suggesting that TQ might induce U251R cell apoptosis through p‐38 activation (Figure 4B).

The anticancer effects of TQ, TMZ, and TQ and TMZ cotreatment were further evaluated using an animal xenograft model involving U251R cells. TQ treatment and TQ and TMZ cotreatment significantly reduced the xenograft tumor volumes, whereas no significant differences were observed between the control and TMZ treatments (Figure 5A). Furthermore, MGMT expression was not significantly different between groups, hinting that neither TQ nor TMZ effectively alter MGMT expression in U251R cells. TQ is found to induce apoptosis, and this phenomenon might be responsible for its genotoxicity (DNA fragmentation assessed by TUNEL and DAPI).^29^, ^30^, ^31^, ^32^ TUNEL‐DAPI staining confirmed that TQ alone and TQ combined with TMZ caused apoptosis in xenograft tumors (Figure 5B). Protein expression results were similar to those obtained in vitro with U251R cells. The p‐p38 and cleaved caspase‐3 levels increased following treatment with TQ or a combination of TQ and TMZ (Figure 5C).

Several reports have indicated that TMZ is hydrolyzed and converted into methyl triazeno imidazole carboxamide (MTIC) when the cellular pH is lower than pH 7.4; MTIC can affect GBM cells through its DNA alkylating effect.^33^, ^34^ This is the likely reason why HPLC did not detect TMZ in the brain tissues of nude mice. Recently, TQ has also been reported to degrade in strongly acidic (0.1 M HCl) or strongly basic (0.1 M NaOH) environments; however, it is relatively stable under the normal physiological conditions present within cells.^35^ TQ prepared in normal saline (pH 7.4)—for preventing TQ degradation—was administered through IP. Although several studies have shown that TQ treatment can protect neurons from inflammation or oxidative stress in vivo, evidence that TQ can pass through the BBB is still lacking.^36^, ^37^

Furthermore, TQ concentrations in murine brain tissue samples and different concentrations of TQ standard solutions (for calibration curves) have been analyzed by HPLC assays.^21^, ^38^ We found that the TQ concentration in the mouse brain tissue was 25 ± 4 μM, which suggests that TQ can pass through the BBB in the murine model (Figure 6).

Thymoquinone exhibits antitumor functions through the promotion of apoptosis, arrest of cell cycle, inhibition of angiogenesis and metastasis in cancer.^39^ Recently, additional mechanisms, including cell apoptosis, necrosis, and autophagy, by TQ alone or TQ cotreatment with gemcitabine, have been reported in both MCF‐7 (human breast adenocarcinoma) and T47D (human ductal carcinoma) cell lines.^40^ Similarly, TQ treatment may induce cell autophagy by increasing the LC3‐II/LC3‐I ratio and Beclin‐1 expression rather than direct induction of apoptosis in the docetaxel‐resistant human prostate cancer PC3 cell line.^41^ A possible mechanism of TQ‐induced non‐apoptotic death is through an increase in endoplasmic reticulum stress and its associated pro‐necroptotic effect. This presents TQ‐selective therapeutic relevance compared with standard chemotherapy agents in diffuse large B cell lymphoma (DLBCL) therapies.^42^ The antitumor ability of TMZ, as a standard chemotherapy agent for GBM, can be attributed to DNA alkylation in GBM cells, which eventually leads to cell cycle arrest in G2/M and apoptosis.^17^ Therefore, we hypothesized that TQ + TMZ cotreatment would have a more significant effect; however, the actual results showed that the effects of TQ alone and TQ + TMZ were nearly the same. A possible reason is that the antioxidant capacity of TQ affects the therapeutic effect of TMZ. Future studies should use different procedures or different dosage forms to enhance the effects of the TQ + TMZ treatment.

In conclusion, our results showed that TQ can penetrate the BBB and exhibits a promising anti‐GBM effect. Compared with the current clinical drug TMZ, TQ is not disturbed by the crosstalk between ERK and MGMT. Furthermore, used alone or in combination with TMZ, TQ enhanced the apoptosis of TMZ‐resistant cells. Among the few available GBM drug classes, TQ molecules show good therapeutic potential. However, more careful safety assessment and pharmacokinetic testing are still needed before the use of TQ for the clinical treatment of GBM. The TQ molecule itself or a molecule made structurally similar to TQ after chemical modification may serve as a potential drug for the treatment of GBM.

## AUTHOR CONTRIBUTIONS

Ai Mai, Shu‐Wen Ye, and Jia‐Yu Tu: conception and design, interpretation of data; Jun Gao and Zhan‐Fang Kang: acquisition and analysis of data, drafting the manuscript; Shu‐Wen Ye and Wei‐Jen Ting: conception and design, given final approval of the version to be published; Qian‐Ming Yao and Wei‐Jen Ting: analysis and interpretation of data, drafting and revising the manuscript. All authors have read and approved the manuscript.

## CONFLICT OF INTEREST

The authors declare no conflicts of interest.
